# HTLV-1 bZIP factor suppresses c-Fos transcription and impairs T cell activation

**DOI:** 10.1186/1742-4690-11-S1-P92

**Published:** 2014-01-07

**Authors:** Akihiro Kawatsuki, Jun-ichiro Yasunaga, Masao Matsuoka

**Affiliations:** 1 Laboratory of Virus Control, Institute for Virus Research, Kyoto University, Kyoto, Kyoto, Japan

## 

c-Fos forms AP-1 heterodimer with JUN family proteins such as c-Jun and functions as a transcription factor to regulate diverse biological processes including T cell activation. Although an HTLV-1-encoded oncoprotein Tax induces the expression of c-*fos*, its expression is significantly down-regulated in fresh ATL cells compared with CD4+ T cells of healthy donors. In this study, we found that HTLV-1 bZIP factor (HBZ) is responsible for the suppressed *c-fos* transcription in ATL cells. The results of reporter assay implied the roles of LXXLL motif of HBZ on the suppression of *c-fos*. HBZ has been reported to suppress AP-1 and NFAT signaling pathways through the direct interaction with c-Jun and NFATc2, respectively. We found c-Fos overexpression impairs the suppressive effects of HBZ on AP-1 and NFAT, suggesting that HBZ overcomes the inhibitory effects of c-Fos by suppressing its transcription. HBZ is known to bind to c-Jun instead of c-Fos. Suppressed transcription of *c-fos* facilitates HBZ to interact with c-Jun, and enhances suppressive effect of HBZ on AP-1 pathway. AP-1 and NFAT signaling pathways are activated by T-cell receptor (TCR) stimulation, leading to T cell activation. We found that TCR stimulation induces the *c-fos* up-regulation and the expression of the activation marker CD69 on CD4+ T cells of wild type mice, but not of HBZ transgenic mice, indicating that the activation of the signaling pathways initiated from TCR are suppressed by HBZ. We hypothesize that *c-fos* suppression by HBZ may contribute to impaired activation of T cells and pathogenesis of HTLV-1 infection.

